# Antiarrhythmic Effects of Melatonin and Omega-3 Are Linked with Protection of Myocardial Cx43 Topology and Suppression of Fibrosis in Catecholamine Stressed Normotensive and Hypertensive Rats

**DOI:** 10.3390/antiox9060546

**Published:** 2020-06-22

**Authors:** Barbara Szeiffova Bacova, Csilla Viczenczova, Katarina Andelova, Matus Sykora, Kiranj Chaudagar, Miroslav Barancik, Michaela Adamcova, Vladimir Knezl, Tamara Egan Benova, Peter Weismann, Jan Slezak, Narcisa Tribulova

**Affiliations:** 1 Centre of Experimental Medicine, SAS, 84104 Bratislava, Slovakia; barbara.bacova@savba.sk (B.S.B.); viczencz.csilla@gmail.com (C.V.); katarina.andelova@savba.sk (K.A.); matus.sykora@savba.sk (M.S.); miroslav.barancik@savba.sk (M.B.); vladimir.knezl@savba.sk (V.K.); tamara.benova@savba.sk (T.E.B.); jan.slezak@savba.sk (J.S.); 2 Research Center for Molecular Medicine of the Austrian Academy of Sciences, A-1090 Vienna, Austria; 3 L. M. College of Pharmacy, Ahmedabad, Gujarat 380009, India; kiranjcology@gmail.com; 4 Department of Physiology, Faculty of Medicine, Charles University, 50003 Hradec Kralove, Czech Republic; adamcova@lfhk.cuni.cz; 5 Faculty of Medicine, Comenius University, 81499 Bratislava, Slovakia; peter.weismann@fmed.uniba.sk

**Keywords:** rat heart, isoproterenol, connexin-43, extracellular matrix, ventricular fibrillation, melatonin, omega-3 fatty acids

## Abstract

Cardiac β-adrenergic overstimulation results in oxidative stress, hypertrophy, ischemia, lesion, and fibrosis rendering the heart vulnerable to malignant arrhythmias. We aimed to explore the anti-arrhythmic efficacy of the anti-oxidative and anti-inflammatory compounds, melatonin, and omega-3, and their mechanisms of actions in normotensive and hypertensive rats exposed to isoproterenol (ISO) induced β-adrenergic overdrive. Eight-month-old, male SHR, and Wistar rats were injected during 7 days with ISO (cumulative dose, 118 mg/kg). ISO rats were either untreated or concomitantly treated with melatonin (10 mg/kg/day) or omega-3 (Omacor, 1.68 g/kg/day) until 60 days of ISO withdrawal and compared to non-ISO controls. Findings showed that both melatonin and omega-3 increased threshold current to induce ventricular fibrillation (VF) in ISO rats regardless of the strain. Prolonged treatment with these compounds resulted in significant suppression of ISO-induced extracellular matrix alterations, as indicated by reduced areas of diffuse fibrosis and decline of hydroxyproline, collagen-1, SMAD2/3, and TGF-β1 protein levels. Importantly, the highly pro-arrhythmic ISO-induced disordered cardiomyocyte distribution of electrical coupling protein, connexin-43 (Cx43), and its remodeling (lateralization) were significantly attenuated by melatonin and omega-3 in Wistar as well as SHR hearts. In parallel, both compounds prevented the post-ISO-related increase in Cx43 variant phosphorylated at serine 368 along with PKCε, which are known to modulate Cx43 remodeling. Melatonin and omega-3 increased SOD1 or SOD2 protein levels in ISO-exposed rats of both strains. Altogether, the results indicate that anti-arrhythmic effects of melatonin and omega-3 might be attributed to the protection of myocardial Cx43 topology and suppression of fibrosis in the setting of oxidative stress induced by catecholamine overdrive in normotensive and hypertensive rats.

## 1. Introduction

Increased levels of circulating catecholamine under stressful conditions, including post-infarction, provide a stimulus for the development of both cardiac hypertrophy and myocardial cell injury. The adverse actions of catecholamines may result from the direct overstimulation of α- and β-adrenoceptors and the indirect effects elicited by the formation of oxidation products, such as adrenochromes and oxyradicals [1,2]. Subsequently, myocardial hypertrophy, ischemia, and oxidative stress accompanied by intracellular Ca^2+^-overload result in cardiomyocyte damage. Myocardial injury facilitates the development of malignant arrhythmias and hence increase the risk for sudden cardiac death (SCD) [1,3,4,5]. 

Rodents exposed to a synthetic catecholamine, isoproterenol (ISO), is an established model for studying structural, subcellular, electrophysiological, and functional alterations in the heart during the development of stress-induced cardiomyopathy [1,6,7,8]. A higher dose of ISO is used as a non-invasive myocardial infarction model implicating oxidative stress, ischemia, inflammation, infarct-like necrosis, and replacement fibrosis [8,9,10,11,12,13]. These events may affect the expression of electrical coupling protein connexin-43 (Cx43), thereby promoting arrhythmias [14,15]. However, the impact of Cx43 and extracellular matrix (ECM) alterations on the susceptibility of the heart to life-threatening arrhythmias has not been thoroughly explored in these conditions.

In this context, it should be emphasized the critical role of catecholamine oxidation and the oxidative stress in the genesis of malignant ventricular arrhythmias associated with various stressful conditions [1,16,17]. Reactive oxygen species (ROS) can virtually damage cellular components and affect essential biological processes, including protein synthesis and translational regulation. Notably, oxidative stress deteriorates Cx43 expression that may affect myocardial conduction [16,18]. ROS along with matrix metalloproteinases (MMPs) have been implicated in the development of cardiac fibrosis [19], which disturbs electrical wave propagation promoting myocardial instability. These factors determine the propensity for cardiac arrhythmias [20].

On the other hand, approaches aimed to prevent or attenuate oxidative stress have been shown cardioprotective. Recent studies suggest that targeting arrhythmogenic redox imbalance may provide novel therapeutics to treat or prevent life-threatening arrhythmias and SCD [3,16]. In harmony with it, a large body of evidence of experimental studies point out the benefit of pineal hormone melatonin due to its pleiotropic anti-oxidant and anti-inflammatory actions [11,21,22,23]. Arrhythmias and abnormalities of Cx43 can be prevented predominantly by mitochondria-targeted antioxidants and the property of melatonin is that it can easily penetrate cells and mitochondria, where it can eliminate the excess of free radicals [24].

Similarly, anti-inflammatory and anti-oxidant actions of omega-3 most likely underlie their cardioprotective and anti-arrhythmic properties [25]. Purified omega-3, rich in eicosapentaenoic acid (EPA) and docosahexaenoic acid (DHA) such as Omacor, Avaza, Vascazen, Omazen, etc., cannot be considered only as nutritional adjuncts because they exert drug-like properties. Compared to drugs, the undesired side effects of omega-3 are rare. There are numerous mechanisms, by which circulating and incorporated omega-3 may act at the cellular and molecular levels, including genetic and epigenetic modulations [25,26].

Taken together, we hypothesized that the mentioned compounds might be efficient in preventing potentially lethal arrhythmias in catecholamine-induced stressful conditions. If so, it would support the rationale to monitor endogenous levels of melatonin and omega-3, particularly in high-risk individuals as well as in patients suffering from coronary heart diseases. Therefore, we aimed to explore the anti-arrhythmic potential of melatonin and omega-3 along with the implication of their downstream targets, Cx43 and ECM proteins, in normotensive and hypertensive rats exposed to catecholamine overdrive.

## 2. Materials and Methods 

### 2.1. Experimental Design

Recommendations in the Guide for the Care and Use of Laboratory Animals published by US National Institutes of Health (NIH publication No. 85-23, revised 1996) which are in agreement with the Animal Health and Animal Welfare Division of the State Veterinary and Food Administration of the Slovak Republic were strictly respected throughout the whole animal experiment. Rats were housed in plastic cages with an ensured temperature of 23 ± 1 °C, 12-h light/dark cycles, ad libitum access of tap water and standard laboratory chow.

**Figure 1:**
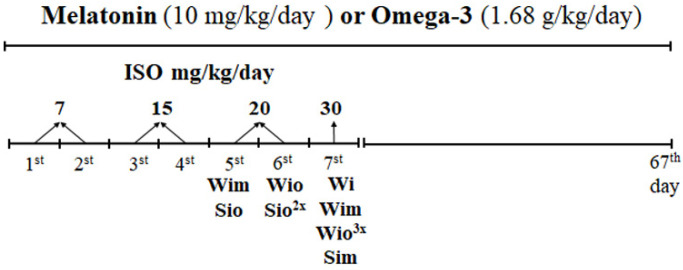
Design of the experiment and mortality rate: ISO was applied during 7 days in dose 7, 15, 20, 30 mg/kg (s.c.) to S and W rats. Drinking of melatonin and feeding with omega-3 was realized from the 1st until the 67th day. The highest mortality was registered after the last injection of ISO on day 7. W-Wistar rats; Wi-isoproterenol injured W; Wim-melatonin treated Wi; Wio-omacor treated Wi; S-spontaneously hypertensive rats; Si-isoproterenol injured S; Sim-melatonin treated Si; Sio-omacor treated Si.

In the experiments, we used 8-month-old male Wistar rats (W, *n* = 64) and age-matched spontaneously hypertensive rats (S, *n* = 64) which were randomly divided into Isoproterenol (ISO)-treated and non-treated groups. Isoproterenol hydrochloride (Sigma-Aldrich, St. Louis, MI, USA) was dissolved in saline and injected subcutaneously for 7 days in a graded manner from 7 mg/kg to 30 mg/kg. Besides that, rats were supplemented with melatonin (N-Acetyl-5-methoxytryptamine) served in drinking water at night time (M5250, Sigma-Aldrich, USA, 40 µg/mL/day) or omega-3 fatty acids by gastric tube (Omacor, Pronova Biopharma Norge AS, Sandefjord, Norway, 1.68 g/kg/day) for 67 days (Figure 1). 

Melatonin was dissolved in drinking water and its appropriate dosage was calculated to the water consumption of one rat. Melatonin protection from light was assured by black bottle usage.

Omega-3 were obtained from capsules which each contain 1000 mg of omega-3-acid ethyl esters (460 mg eicosapentaenoic acid ethyl ester and 380 mg docosahexaenoic acid ethyl ester), and 4 mg d-alpha-tocopherol as an antioxidant. According to our previous experimental studies, melatonin [21,27,28] and omega-3 PUFA [26,29,30,31], protect hypertensive, hyperthyroid, hypertriglyceridemic, and aged rats from malignant arrhythmias.

At the end of the experiment, systolic blood pressure using tail-cuff plethysmography through Statham Pressure Transducer P23XL (Hugo Sachs, March-Hugstetten, Germany) was registered. Finally, rats were injected with anesthetic ketamine (Narketan; Vetoquinol UK Ltd., Towcester, UK, 100 mg/kg) and myorelaxant xylazine (Xylapan; Vetoquinol UK Ltd., UK, 10 mg/kg). After thoracotomy, hearts were excised, rinsed with ice-cold physiological saline, and used for further experiments. Subsequently, samples of retroperitoneal and epidydimal adipose tissue were taken and weighed.

### 2.2. Examination of Heart Function and Ventricular Fibrillation in Langendorff-Perfused Hearts

An ex vivo Langendorff-mode perfused rat heart, as previously described [21], was used for an examination of heart function and ventricular fibrillation (VF). Very briefly, the hearts of 6 rats from each group were perfused via cannulated aorta in Langendorff mode with oxygenated Krebs–Henseleit solution (mmol/L: 120 NaCl; 4.2 KCl; 1.75 CaCl_2_; 1.25 MgSO_4_.4H_2_O; 12.5 glucose; 25.0 NaHCO_3_, pH = 7.4) at a constant pressure of 80 mmHg (1 mmHg = 133.322 Pa) and temperature (37.0 ± 0.3 °C). After 20 min of the heart equilibration and recording of basal hemodynamic parameters, induction of sustained VF (lasting > 2 min) by electrical stimulation via electrodes located at epicardium of the right ventricle was performed. One second burst of electrical rectangular pulses at a 10 mA current strength (Electrostimulator ST-3″, Medicor, Illatos, Hungary) was used. In case sustained VF was not induced, the stimulus intensity was increased in 5 mA steps until a maximum of 50 mA [21].

### 2.3. SDS-PAGE and Western Blot Analysis 

As described previously [32], frozen left ventricular heart tissue was homogenized in lysis buffer (20% SDS, 10 mmol/L EDTA, 100 mmol/L Tris, pH 6.8) and diluted in Laemmli sample buffer under reducing and non-reducing conditions. Loading equal amounts of protein per lane were separated in 10% SDS-polyacrylamide gels at a constant voltage of 120 V (Mini-Protean TetraCell, Bio-Rad, Hercules, CA, USA). Electrically transferred proteins to a nitrocellulose membrane (0.2 m pore size, Advantec, Tokyo, Japan) were blocked for 4 h with 5% low-fat milk and then incubated overnight at 4 °C with primary antibodies (Table 1). After washing three times with Tris-buffered saline with 0, 1% Tween (TBS-T), membranes were incubated for 1 h with a horseradish peroxidase-linked secondary antibody (anti-rabbit, diluted 1:2000, Cell Signaling Technology, Danvers, MA, USA, #7074/; anti-goat, diluted 1:10000, Sigma-Aldrich, St.Louis, MO, USA #A5420), 6 times washed in TBS-T and visualized by enhanced chemiluminescence method. Bands were quantitated by densitometric analysis using Carestream Molecular Imaging Software (version 5.0, Carestream Health, New Haven, CT, USA).

### 2.4. Zymographic Detection of MMP-2 Activity

Left ventricular tissue was homogenized and diluted as in the western blot method in non-reducing conditions. Samples were loaded (40 µg protein/lane) into wells of 10% gels copolymerized with gelatin (2 mg/mL) (Mini-Protean TetraCell, Bio-Rad) and separated by SDS-PAGE. Gels were then 2 times washed for 30 min with washing buffer at room temperature (50 mmol/L Tris-HCl, 2.5% Triton X-100, pH 7.4) and incubated overnight in developing buffer at 37 °C (50 mmol/L Tris-HCl, 10 mmol/L CaCl_2_, 1.25% Triton X-100, pH 7.4). Subsequently, the gels were stained for one hour with stain solution (1% Coomassie Brilliant Blue G-250 dissolved in a solution containing 10% acetic acid and 40% methanol). Finally, were gels destained with a solution containing 10% acetic acid and 40% methanol until a clear resolution between the transparent bands and dark blue background appeared. Transparent clear bands were evaluated as enzymatic activities of MMP-2 using Carestream Molecular Imaging Software (version 5.0, Carestream Health, New Haven, CT, USA) [33].

### 2.5. Immunofluorescence of Cx43 and Quantitative Image Analysis

As previously described [21], for immunodetection of Cx43 distribution we used 10 µm thick left ventricular cryosections. Tissue sections were washed in phosphate buffer saline (PBS), fixed in ice-cold methanol, permeabilized in 0.3% Triton X-100 in PBS, and blocked with the solution of 1% bovine serum albumin in PBS. Overnight incubation at 4 °C with primary anti-Cx43 antibody (diluted 1:500, CHEMICON International, Inc., Temecula, CA, USA, #MAB 3068) was followed by PBS washes and 2-h incubation with FITC-fluorescein isothiocyanate (diluted 1:500, Jackson Immuno Research Labs, West Grove, PA, USA, # 111-095-003). After several times of PBS washes, tissues were mounted in the Vectashield medium (H-1200, Vector Laboratories-Inc., Burlingame, CA, USA) and captured by Zeiss Apotome 2 microscope (Carl Zeiss, Jena, Germany).

For quantification of Cx43 labeling, a randomly selected area (15 per heart) of positive immunofluorescence signal were analyzed and defined as a number of pixels with the Cx43 signal intensity exceeding a threshold of 30 on the 0–255 Gy scale. The total number of Cx43 positive pixels was expressed as a total integral optical density per area (IOD) [34]. For quantification of Cx43 lateralization, a manual selection and delineation of terminal intercalated disc-related areas of Cx43 were performed in the 7 test field per slice [28]. The difference between total IOD per area and IOD of terminal areas corresponds to the IOD of lateral Cx43 immunofluorescence signal (Image-Pro Plus). The lateral Cx43 topology was expressed as a percentage calculated from the ratio of IOD of lateral topology divided by the total IOD. The values were statistically compared among the groups.

### 2.6. Determination of Collagen Content by Hydroxyproline Measurement

Hydroxyproline content in the left ventricle tissue samples as a marker of fibrosis was estimated using the spectrophotometric method as described previously [35,36]. Samples were hydrolyzed in 6 M HCl for 3 h at 130 °C. Dried samples were treated at room temperature with chloramine T in the acetate-citrate buffer (pH 6.0). The oxidation reaction was stopped after 20 min by adding Ehrlich’s reagent solution. After the samples incubation at 65 °C for 15 min, the concentration of hydroxyproline was measured spectrophotometrically at 550 nm. The hydroxyproline content was then expressed in mg per total weight of the LV.

### 2.7. Determination of Collagen Deposition by van Gieson Technique and Quantitative Image Analysis

For examination of collagen deposition, 10 µm thick left ventricular cryosections were stained according to the van Gieson technique as previously described [29]. Tissue samples were fixed with 4% buffered formaldehyde solution, washed in PBS and stained for 5 min with a mixture of a saturated aqueous solution of picric acid with 1% aqueous solution of acid fuchsine. Finally, the sections were washed with acidified water and examined in a light microscope (Axiostar, Carl Zeiss, Jena, Germany) followed by images acquisition.

The red staining of collagen deposition was clearly recognized on the background of the yellow stained heart cardiomyocytes. 6 images per heart were analysed by Soft Imaging System GmBH (Münster, Germany). Collagen staining was expressed as a percentage of the total tissue area.

### 2.8. Determination of Alkaline Phosphatase Activity and Quantitative Image

The capillary related activity of alkaline phosphatase (E.C.3.1.3.1) was determined according to the method of Lojda and Gutmann (1976) [37]. 10 µm thick cryostat sections were incubated in buffered medium containing sodium β-glycerophosphate as a substrate, magnesium sulphate as an activator, and calcium chloride for precipitation of released phosphate. Calcium phosphate was visualized by reaction with yellow ammonium sulfide and examined using a light microscope (Axiostar; Carl Zeiss Jena, Germany). Acquired microscopic images (15 per heart) with a colored histochemical reaction (dark brown) were analyzed by Soft Imaging System, GmbH (Germany) as previously reported [34]. The signal was expressed as a percentage of the total tissue area.

### 2.9. Determination of Succinate Dehydrogenase and β-Hydroxybutyrate Dehydrogenase Activities

The activity of mitochondrial enzymes succinate dehydrogenase (SDH, EC 1.3.5.1) and β-hydroxybutyrate dehydrogenase (b-HBDH, EC 1.1.1.30) that are components of the respiratory chain involved in energetic metabolism, were determined according to Lojda and Gutmann (1976) [37]. 10 µm thick cryostat sections were incubated for 45 min at 37 °C in a phosphate-buffered solution containing succinate or β-hydroxybutyrate as a substrate, NAD as a coenzyme and nitroblue tetrazolium chloride as a hydrogen acceptor. The blue colored final reaction product was examined in a light microscope (Axiostar; Carl Zeiss Jena, Germany).

### 2.10. Measurement of Lipoperoxidation by Thiobarbituric Acid Reactive Substances (TBARS) Assay

TBARS levels were analyzed, as a marker of oxidative stress and lipoperoxidation, according to Shlafer and Shepard (1984) [38] with modifications [39]. 40 µL of tissue homogenates and 40 µL of 20% trichloroacetic acid solution were mixed with a 320 µL of TBARS reagent (37 mmol/L C4H_4_N_2_O^2^S, 500 mmol/L NaOH, 15% *v*/*v* CH_3_COOH) and incubated for 70 min at 100 °C. Subsequently, the mixture was cooled on ice for 10 min, pipetted into another tube with a mixture of *n*-butanol and pyridine (14:1, *v*/*v*), and centrifuged 10 min at 5000× *g*. The organic phase was used for measurement of absorbance at 535 nm by Synergy H1 Hybrid Multi-Mode Microplate Reader (Biotek, Vermont, USA). The concentration of malondialdehyde (MDA) in the samples was calculated according to a calibration curve made from tetrabutylammonium malondialdehyde salt.

### 2.11. Statistical Analysis

Differences between groups were evaluated using one-way ANOVA and Tukey’s multiple comparison tests. Kolmogorov–Smirnov normality test to examine if variables are normally distributed was used. Data were expressed as mean ± SD; *p* < 0.05 was considered to be statistically significant.

## 3. Results

### 3.1. Survival of Experimental Rats and Registered Biomarkers

Stepwise increase of ISO dose during one week resulted in the death of one Wistar rat, while none of SHR. Unexpectedly, the concomitant administration of ISO with omega-3 resulted in the death of 4 Wistar rats and 3 SHR whereby mortality was prevalent on the 7th day of ISO application (see Figure 1). Concomitant administration of ISO with melatonin resulted in the death of 1 SHR and 2 Wistar rats. Findings suggest the risk of adverse acute interaction of beta-adrenergic overstimulation with omega-3 and to a lesser extent with melatonin, probably due to mutual adrenergic and redox burden, whereby the overall mortality was lower in hypertensive versus normotensive rats.

As shown in Table 2, blood pressure was higher and bodyweight lower in SHR versus Wistar rats, and these parameters were not significantly affected either by ISO administration or by treatment with melatonin and omega-3. There was a mild increase of heart weight in both strains registered 60 days upon ISO withdrawal. In parallel, a moderate but significant increase of left ventricular weight was registered in Wistar rats, whereby it was prevented by the treatment of either compound. There was no increase of left ventricular weight in SHR 60 days after response to ISO.

### 3.2. Heart Function and VF Threshold Registered Ex Vivo

Coronary flow as well as indexes of contractility and relaxation were significantly increased in Wistar and SHR hearts registered 60 days upon ISO withdrawal when comparing to the strain-matched controls, while heart rate did not change. ISO-induced changes reflecting enhanced heart function during the adaptation period persisted in omega-3 or melatonin treated groups in both rat strains (Table 3).

**Figure 2:**
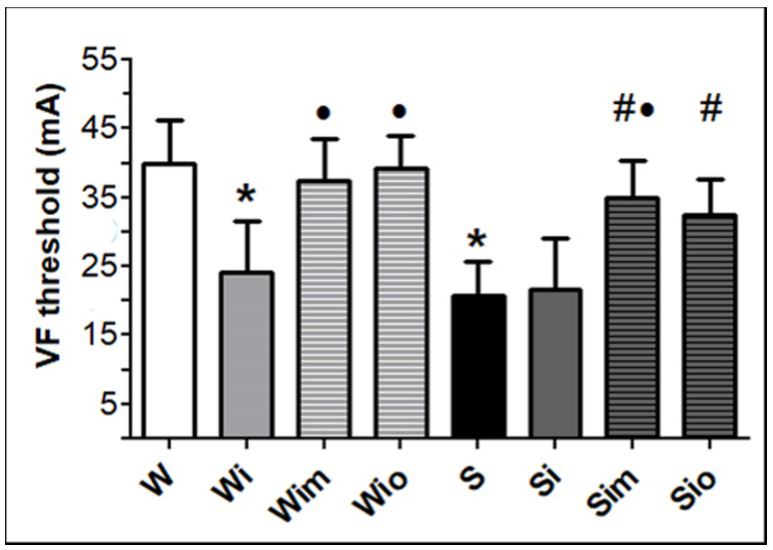
The threshold to induce persistent VF was lower in spontaneously hypertensive rat hearts (S) compared to Wistar rat (W) heart and reduced in W rats in response to ISO (Wi) while not in S. Treatment of ISO exposed rats with either melatonin (Wim, Sim) or omega-3 resulted (Wio, Sio) in a significant increase of VF threshold in both strains. Values are the mean ± SD of 6 rats in each group. * *p* < 0.05 compared with W; ^#^ *p* < 0.05 compared with S; ^•^ *p* < 0.05 compared to Wi vs. Wim, Wio/Si vs. Sim, Sio.

The threshold to induce sustained VF (Figure 2) was significantly lower in SHR compared to Wistar rat hearts. The decline of the VF threshold was registered 60 days of the post-ISO period but only in Wistar rats. In contrast, the treatment with melatonin or omega-3 increased the VF threshold in Wistar rats.

### 3.3. Cardiomyocyte Alterations in Left Heart Ventricle

**Figure 3:**
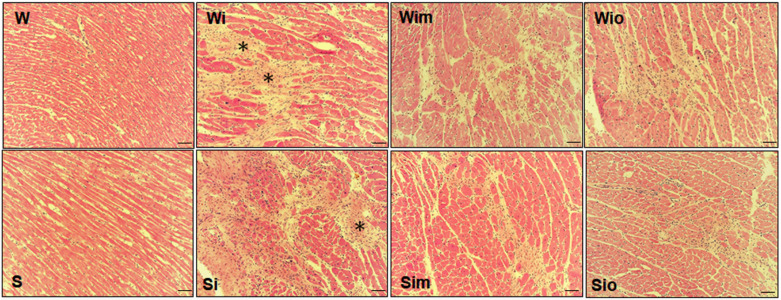
Diffuse ISO-induced myocardial injury characterized by necrotic and atrophied cardiomyocytes (asterisks) as well as by infiltration with basophils in Wistar rats (Wi) and Spontaneously hypertensive rats (Si) comparing to controls (W, S). Note reduced foci of injury after treatment of ISO exposed rats to melatonin (Wim, Sim) or omega-3 (Wio, Sio). Haematoxylin-eosin staining, bar 200 μm.

Hematoxylin-eosin staining of ISO affected Wistar and SHR hearts revealed that the prevalent population of cardiomyocytes did not differ from control rat hearts. Apart from it, there were patchy areas of myocardial injury (Figure 3) consisting of the population of the hypertrophied and atrophied/necrotic cardiomyocytes surrounded by altered extracellular space richly infiltrated with neutrophils and macrophages. Myocardial injury was suppressed by treatment with melatonin and omega-3 in both strains as indicated by smaller foci of severely altered tissue (Figure 3).

**Figure 4:**
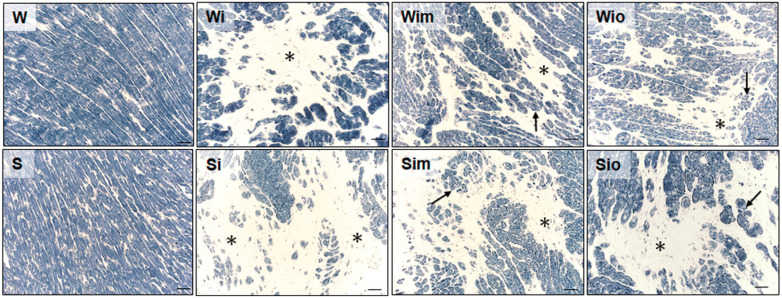
Reliable histochemical demonstration of SDH activity revealed its absence in necrotic cardiomyocytes (asterisks) and reduced activity in surrounding atrophied myocytes in ISO-exposed Wistar (Wi) and Spontaneously hypertensive rat (Si) hearts. Note apparent preservation of SDH activity in viable cardiomyocytes (arrows) and reduced areas of injury (asterisks) in ISO-exposed rats after treatment with melatonin (Wim, Sim) or omega-3 (Wio, Sio). The intensity of staining in controls (W, S) corresponds to normal SDH activity. Bar 200 μm.

Catalytic enzyme histochemistry revealed high activity (corresponding with the high intensity of staining) of SDH and bHBDH (not shown) in the majority of the cardiomyocytes of ISO affected rat hearts regardless of the strain (Figure 4). Moreover, the activity of both enzymes (shown only SDH) was decreased but still maintained in viable cardiomyocytes located in the areas of myocardial injury. Treatment of ISO-affected rats with melatonin and omega-3 preserved activities of both enzymes involved in mitochondrial energetic metabolism even in the viable cardiomyocytes at injured areas, see a demonstration of SDH in Figure 4.

**Figure 5:**
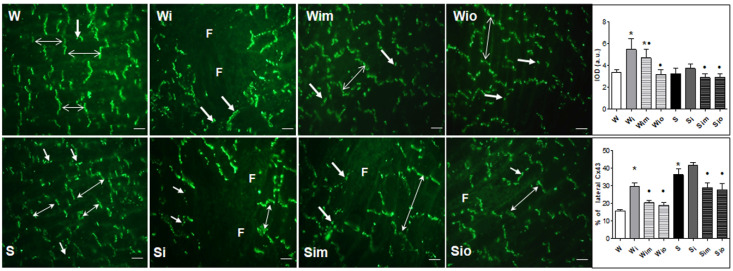
The myocardial topology of Cx43 immunofluorescence labeling. Note absence of Cx43 in areas of injury (F) and disordered distribution of Cx43 in surrounding viable cardiomyocytes in ISO-exposed heart (Wi, Si) compared to controls (W) with a regular polar distribution of Cx43 confined to intercalated discs (long arrows) and lateral sides of the cardiomyocytes (short arrows) that is more frequent in Spontaneously hypertensive rat (S) hearts. Abnormal distribution of Cx43 was attenuated by treatment with melatonin (Wim, Sim) and omega-3 (Wio, Sio). Quantitative image analysis revealed enhanced Cx43 positive signals in ISO-exposed rats that were suppressed by treatment (upper graphs). Importantly, the treatment of ISO exposed rats by melatonin and omega-3 significantly suppressed laterally distributed Cx43 (bottom graphs). * *p* < 0.05 compared with W; ^•^ *p* < 0.05 compared to Wi vs. Wim, Wio/Si vs. Sim, Sio. Bar 100 um.

Specific Cx43 immunolabeling revealed its enhanced localization at the lateral sides of the cardiomyocytes (in addition to conventional polar distribution at the intercalated discs located gap junctions) in ISO-affected rat hearts of both strains (Figure 5). Moreover, hypertrophied and atrophied cardiomyocytes in the focal areas of myocardial injury exhibited pronounced mislocalization of Cx43. An overall abnormal pattern of Cx43 distribution was attenuated by treatment with melatonin and omega-3 regardless of the strain as identified by quantitative image analysis. In particular, treatment significantly suppressed the ISO-induced increase of the Cx43 at the lateral sides of the cardiomyocytes in both rat strains (Figure 5). The increase of the total Cx43-positive signal in response to ISO was blunted by treatment as well.

**Figure 6:**
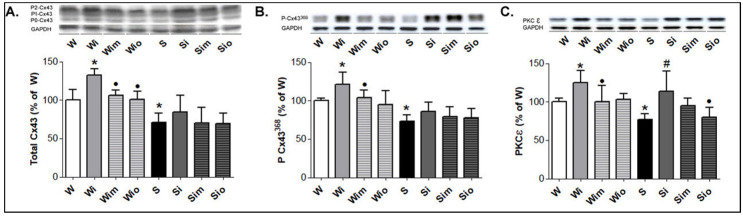
Western blot demonstration of Cx43 protein levels (**A**), its functional phosphorylated forms (**B**) and PKCε protein levels (**C**) in experimental rats. Note reduced levels of Cx43 in S compared to W rats and increase of Cx43 (*p* < 0.05 in W) along with PKCε in ISO-stressed rats of both strains. In contrast, treatment by melatonin and omega-3 prevented an increase of these parameters in W rat and to a lesser extent in S hearts. W-Wistar rats; Wi-isoproterenol injured W; Wim-melatonin treated Wi; Wio-omacor treated Wi; S-spontaneously hypertensive rats; Si-isoproterenol injured S; Sim-melatonin treated Si; Sio-omacor treated Si. Values are the mean ± SD of 10 rats in each group. * *p* < 0.05 compared with W; ^#^ *p* < 0.05 compared with S; ^•^ *p* < 0.05 compared to Wi vs. Wim, Wio/Si vs. Sim, Sio.

Cx43 protein abundance as well as Cx43 phosphorylated forms at serine 368 were lower in SHR compared to Wistar rats while the increase was detected in both strains after 60 days in response to ISO (Figure 6A,B). Phosphorylated status of Cx43 was linked with the increase of protein abundance of PKCε (Figure 6C). Treatments with either melatonin or omega-3 blunted ISO-induced changes in Cx43 as well as PKCε protein levels in Wistar rat and to a lesser extent in SHR hearts (Figure 6A–C).

### 3.4. Extracellular Matrix Alterations in Left Heart Ventricle

**Figure 7:**
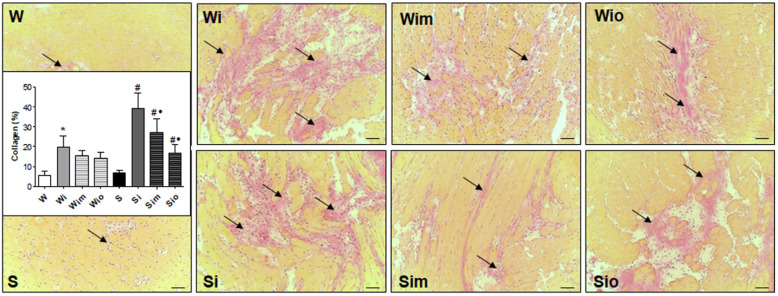
Van Gieson staining demonstrated abnormal collagen deposition (arrows) and a significant increase in ISO-stressed Wistar (Wi) and Spontaneously hypertensive rat hearts (Si) compared to control rat hearts (W, S). The treatment with melatonin (Wim, Sim) and omega-3 (Wio, Sio) suppressed the ISO-induced changes in both strains. * *p* < 0.05 compared with W; ^#^ *p* < 0.05 compared with S; ^•^ *p* < 0.05 compared to Wi vs. Wim, Wio/Si vs. Sim, Sio. Bar 200 μm.

Van Gieson staining was used to differentiate between cardiac myocytes and extracellular collagen followed by light microscopy examination and quantitative image analysis. Representative images (Figure 7) demonstrate the difference in collagen deposition among experimental groups and quantification of deposits. There was an apparent focal increase of collagen-positive staining in Wistar rat as well as SHR hearts 60 days upon ISO withdrawal. On the other hand, treatment with either melatonin or omega-3 attenuated this increase in both ISO-exposed strains.

**Figure 8:**
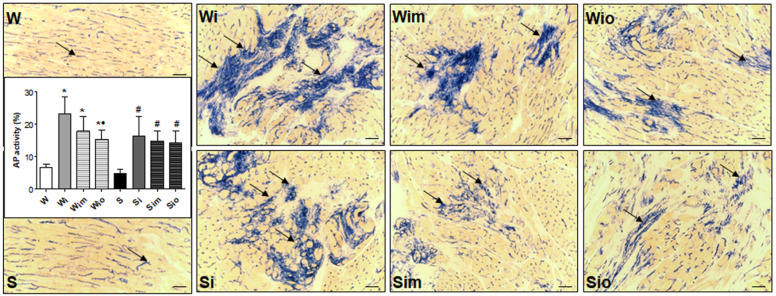
Reliable histochemical demonstration of AP activity (arrows) confined to endothelial cells of capillaries in control Wistar (W) as well as Spontaneously hypertensive rat (S) hearts. In addition, AP activity was increased in the foci of injury in ISO-stressed W rat hearts (Wi) as well as S rat (Si) hearts. Note reduced injured areas with AF activity due to treatment of ISO-stressed rats with melatonin (Wim, Sim) and omega-3 (Wio, Sio). Findings of quantitative image analyses are plotted on graphs. * *p* < 0.05 compared with W; ^#^ *p* < 0.05 compared with S; ^•^ *p* < 0.05 compared to Wi vs. Wim, Wio/Si vs. Sim, Sio. Bar 200 μm.

Catalytic enzyme histochemistry performed on cryostat sections of ventricular tissue revealed that the activity of alkaline phosphatase (AP) is markedly increased in the foci of fibrosis besides its ordinary presence in endothelial cells of capillaries (Figure 8). Data from quantitative image analysis showed that an increase of myocardial AP activity in response to ISO was significant in both strains (more pronounced in Wistar rats) when comparing to control groups. On the other hand, treatment with omega-3 or melatonin significantly attenuated of ISO-induced changes in AP activity in Wistar rat and to a lesser extent in SHR hearts (Figure 8).

**Figure 9:**
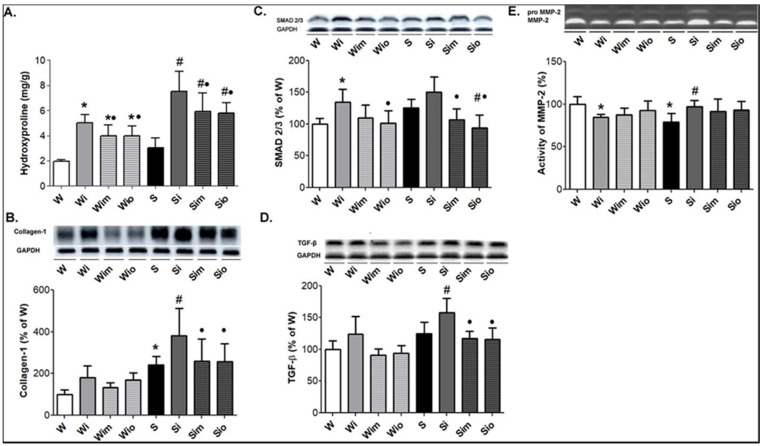
Demonstration of examined hydroxyproline (**A**), collagen-1 (**B**), SMAD2/3 (**C**), and TGF-β (**D**) in experimental rats. There is an increase of hydroxyproline and collagen-1 content paralleled with an increase of SMAD2/3 and TGF-β in ISO-stressed Wistar rat (Wi) and Spontaneously hypertensive rat (Si) hearts. Treatments with melatonin (Wim, Sim) and omega-3 (Sim, Sio) suppressed ISO-induced changes in both strains. Note strain-related differences in ISO actions as well as in effects of treatment. Myocardial MMP-2 activity (**E**) was lower in S compared to W rats and reduced in Wi while increased in Si. Treatment did not alter MMP-2 activity compared to ISO exposed rats (Wi, Si) or controls (W, S). Values are the mean ± SD of 10 rats in each group. * *p* < 0.05 compared with W; ^#^ *p* < 0.05 compared with S; ^•^ *p* < 0.05 compared to Wi vs. Wim, Wio/Si vs. Sim, Sio.

In parallel, the levels of collagen-1 and hydroxyproline content were higher in heart tissue homogenates of SHR then Wistar rats (Figure 9A,B). Exposure of these rat strains to ISO resulted in a significant increase of collagen-1 and hydroxyproline content even 60 days of ISO withdrawn. Treatment with melatonin or omega-3 prevented ISO-induced elevation of collagen-1 and hydroxyproline content in SHR and to a lesser extent in Wistar rats (Figure 9A,B). Protein abundance of SMAD2/3 and TGF-β was slightly higher in SHR compared to Wistar rat hearts (Figure 9C,D). The significant increase of SMAD2/3 levels was identified in Wistar while TGF-β levels in SHR hearts 60 days after ISO burden. Omega-3 and melatonin did suppress significantly both pro-fibrotic factors in ISO-affected SHR hearts while the significance was not reached in ISO-affected Wistar rats (Figure 9C,D).

Gelatinolytic activity of MMP-2 was lower in SHR compared to Wistar rat hearts (Figure 9E). The activity of this matrix metalloproteinase was decreased in Wistar whereas it increased in SHR hearts 60 days after ISO exposure. There was a tendency to normalize MMP-2 activity in ISO-affected rats due to treatment with omega-3 or melatonin regardless of the strain.

**Figure 10:**
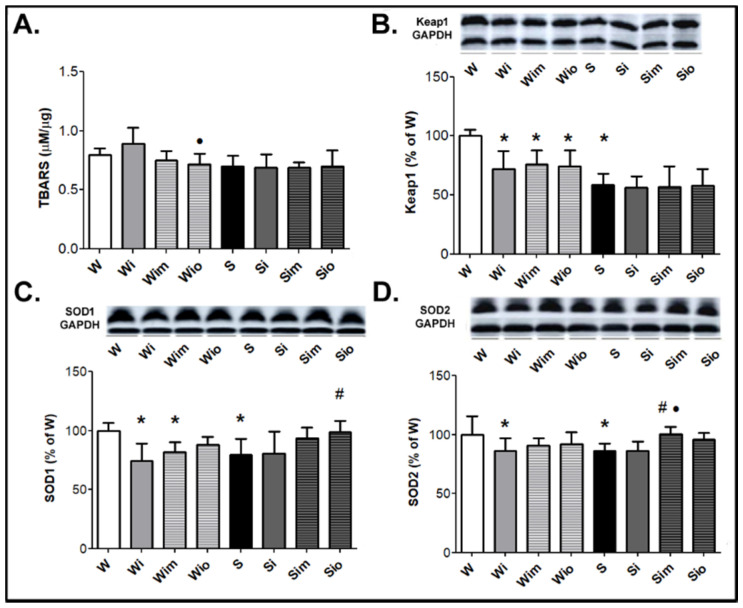
Demonstration of examined myocardial markers modulating redox state, TBARS (**A**), Keap1 (**B**), SOD1 (**C**), and SOD2 (**D**) in experimental rats. There were no changes in TBARS among the groups. The protein level of Keap1 was lower in the spontaneously hypertensive rat (S) compared to Wistar rat (W) hearts and reduced in response to ISO in W rats (Wi) while not in S (Si). Treatment with melatonin and omega-3 did not affect this parameter regardless of the strain (Wim, Wio, Sim, Sio). Protein levels of SOD1 and SOD2 were lower in S compared to W rat hearts. ISO exposure resulted in suppression of SOD isoforms in Wistar rats (Wi) but not in S (Si). There was a tendency to increase SOD1 and SOD2 in both ISO-exposed rat strains by treatment either with melatonin or omega-3. Values are the mean ± SD of 10 rats in each group. * *p* < 0.05 compared with W; ^#^ *p* < 0.05 compared with S; ^•^ *p* < 0.05 compared to Wi vs. Wim, Wio/Si vs. Sim, Sio.

The examination of selected markers of redox status showed that there was no difference in TBARS levels among the experimental groups (Figure 10A). The levels of Keap1 were lower in SHR comparing to Wistar rat hearts, and ISO reduced this parameter in Wistar rats only (Figure 10B). Similarly, the myocardial protein levels of SOD1 and SOD2 were lower in SHR compared to Wistar rat hearts and reduction of responses to ISO was found in Wistar but not in SHR hearts (Figure 10C,D). There was a tendency to increase SOD1 and SOD2 levels in ISO exposed rats with either melatonin or omega-3 treatment in Wistar as well as in SHR hearts (Figure 10C,D).

## 4. Discussion

Maladaptive ventricular remodeling resulting in heart failure (HF) and risk for malignant arrhythmias following acute myocardial infarction (AMI) remains a significant clinical challenge to prevent high mortality [20,40]. Administration of ISO in rodents has been shown to induce cardiac lesions/necrosis, hypertrophy or HF, and death (depending on the dose and duration of application), while cardiac regeneration is limited [7,10,15,41,42]. Similar to the rat heart [11,19,41,43,44,45,46,47], inflammation, oxidative and metabolic stress, as well as tissue remodeling was demonstrated even in human heart slices in response to ISO [13].

To mimic the catecholamine burden which is associated with AMI [40], in this study we used a stepwise increase of ISO dose during seven days application. Only one Wistar rat died after the last dose suggesting that we achieved the border of life-threatening cardiac injury. While, none SHR died due to ISO suggesting that responsiveness to catecholamine overdrive is blunted in this sympathomimetic strain, alike the responses to the excess of thyroid hormones [29]. Strain-related genetic factors have been shown to dominate the susceptibility to ventricular arrhythmias in mice model of β-adrenergic ISO stimulation [48].

To imitate post-infarction arrhythmia prone setting in the clinic [20,40], we focused on the testing of the susceptibility of the heart to arrhythmias in the period when the acute ISO-induced stress is withdrawn, i.e., 60 days post-ISO application. Compared to controls, the threshold to induce VF was reduced in response to ISO in normotensive rats. It supports the impact of catecholamine stress on the development of the pro-arrhythmic setting [1,49]. However, ISO did not affect the VF threshold in hypertensive rat hearts that exhibited significantly lower VF threshold than normotensive rats. It suggests that this rat strain suffering from primary hypertension associated with sympathetic overdrive [27] is less responsive to sudden catecholamine stress. Nevertheless, an increased risk for VF in both strains was associated with enhanced lateralization of Cx43 in hypertrophied cardiomyocytes along with patchy areas of myocardial replacement fibrosis surrounded by mislocalization of Cx43. Such a pro-arrhythmic pattern of Cx43 (see Figure 5) predisposes the disorders of anisotropic conduction and electrical wave propagation promoting electric instability [50,51]. Importantly, lateralization of Cx43 contributed significantly to arrhythmogenesis and lethality in muscular dystrophy mice and young spontaneously hypertensive rats, even though, overall Cx43 levels were increased [14,30]. This suggests that the prevention/attenuation of lateralization of Cx43 may provide substantial benefit.

In the context of arrhythmias, it should be noted that high levels of circulating catecholamines alters Ca^2+^ handling and promotes sarcoplasmic reticulum Ca^2+^-leak and intracellular Ca^2+^-overload [1]. The former facilitates triggered activity and the latter inhibits Cx43 mediated electrical coupling thereby increasing the susceptibility of the heart to arrhythmias [4,52]. Apart from that, persistent activation of the β-adrenergic pathway resulting in cardiac hypertrophy activates pro-arrhythmic Ca^2+/^calmodulin dependent protein kinase II, while its inhibition suppressed cardiac arrhythmias [53].

As pointed out in this and previous studies [54,55,56] in situ Cx43 labeling along with detection of fibrosis and mitochondrial enzymes activity revealed vulnerable myocardial substrate promoting the occurrence of arrhythmias. Notably, the maladaptive Cx43 remodeling (lateralization) may explain the increased susceptibility of the hypertensive and ISO-injured normotensive rat hearts to inducible VF. It is consistent with other arrhythmia prone models accompanied by disorders in Cx43 topology [14,30,54,57,58] that subsequently alters the electrotonic coupling and myocardial conduction [51,59]. Susceptible animals also exhibited enhanced cardiac β-adrenergic responsiveness [60].

Taking into consideration that cardiac adrenoreceptors are directly involved in the control of Cx43-mediated electrical communication [58], they might even be a critical factor affecting regular cell-to-cell conduction and myocardial electrical properties. Even though the β-adrenergic stimulants upregulate Cx43 along with myocardial hypertrophy [58,61]. Consistent with it, an increase of Cx43 protein and its phosphorylation (most likely attributed to enhanced PKCε) persisted 60 days post-ISO treatment. Unfortunately, this increase was linked with enhanced adverse Cx43 lateralization. In addition, ISO exposure resulted in disordered Cx43 distribution that was apparent in hypertrophied and atrophied/necrotic cardiomyocytes surrounding the areas of fibrosis in both normotensive and hypertensive rat hearts.

The process of fibrosis was accompanied by enhanced alkaline phosphatase activity likely due to unfavorable signaling from necrotic myocytes resulting in activation of immune pathways and inflammation [47]. Consequently, loss of cardiomyocytes after infarct-like injury overwhelmed the limited regenerative capacity of the myocardium, resulting in the formation of a collagen-based scar [62]. Indeed, we have shown a significant increase of collagen-positive deposits, collagen-1 abundance, and hydroxyproline content in both normotensive and hypertensive rat hearts in response to ISO. In parallel, MMP-2 activity was increased in hypertensive while not in normotensive rats, as reported by others using the chronic ISO model [6]. Altogether, it is in line with the apparent ISO-induced ECM remodeling which was present regardless of the variabilities of the experimental models. It is worthy to note that the fibrosis persisted (unlike the regression of hypertrophy [63], even after ISO withdrawal as shown in this and other studies [8,15,41,64].

It is of interest that despite pronounced extracellular matrix and Cx43 remodeling the heart function registered ex vivo was preserved in both strains 60 days after ISO withdrawal. The modest improvement in cardiac structure and function during recovery was shown by others, suggesting that ISO injury activates cardiac progenitor cells that can differentiate into new myocytes during cardiac repair [65]. We assume that compensated hypertrophy accompanied by the upregulation of Cx43 allowing metabolic and electrical signals propagation may contribute to the maintenance of heart function during the post-ISO period. The short half-life of Cx43 (~1.5 hrs) indicates the permanent adaptation of cardiomyocyte communication to the actual requirements. Notably, β-adrenergic stimulation enhances this direct Cx43 channel-mediated communication in viable cardiomyocytes [58,61], and even after ISO withdrawal as points out this study. It is consistent with the maintenance of energy production for heart function as indicated by the high activity of mitochondrial SDH and bHBDH. While mitochondrial enzyme activity was reduced after an acute ISO attack [66]. On the other hand, the low dose of ISO administered for 4.5 months did not decrease pump function in normotensive rats [6].

Principal and novel findings of our study deal with the protection of rat hearts against life-threatening arrhythmias by melatonin and omega-3 when applying them during 7 days of ISO application and subsequent 60 days after ISO withdrawal. In normotensive rats, both compounds increased current strength for induction of sustained VF to the level as in control rat hearts. The increase was even more pronounced in hypertensive rats compared to strain-matched controls or the ISO group. Hypertensive rat heart is prone to VF [27] while protection associated with an increase of the VF threshold was afforded by prolonged melatonin treatment [21]. Concern omega-3, the anti-arrhythmic benefit was reported in both experimental and clinical conditions, including myocardial infarction [67]. It should also be emphasized that melatonin and omega-3 exerted acute protection from malignant arrhythmias [22,28,67]. Taken together, this suggests that these compounds may blunt both acute and chronic pro-arrhythmic signaling.

Our study strongly points out the implication of myocardial Cx43 and ECM in melatonin and omega-3 anti-arrhythmic effects. Accordingly, both compounds were powerful to attenuate abnormal Cx43 topology along with the suppression of areas with collagen deposition that was accompanied by a reduced content of hydroxyproline in normotensive as well as in hypertensive rat hearts. ISO-induced ECM remodeling has been shown to be suppressed by various compounds [8,68,69,70] and associated with suppression of the pro-fibrotic SMAD2/3–TGF-β pathway similarly to our study. It appears that melatonin and omega-3 may interfere with signaling pathways facilitating Cx43 and ECM remodeling, whereby free radicals and cytokines might be strong candidates. Oxidative stress is one of the causative factors in pro-arrhythmic signaling [16,43]. In general, redox stress mostly accompanied by inflammation promotes abnormal cardiomyocyte Cx43 distribution and enhances pro-fibrotic signaling [71]. On the other hand, prevention/attenuation of these pro-arrhythmic factors may protect the heart in various pathophysiological conditions, including those induced by catecholamine stress [16,43,72].

Although we have no evidence of anti-oxidant and anti-inflammatory effects of melatonin and omega-3 during the acute stage of ISO effects, there was a clear subsequent benefit from both compounds, including the protection of mitochondria function. Mitochondria respiration rate was shown to be reduced after acute ISO effects but restored by omega-3 [73]. Omega-3 can incorporate into plasma membranes and potentially stabilize them to resist oxidation and confer protection against arrhythmias [67,74]. The levels of tissue lipid peroxidation and reduced glutathione were also brought back to that seen in control animals by continued melatonin treatment [45]. Melatonin, by suppressing the activity of M1 subtype macrophages, reduced inflammation [72] even evoked by autoimmunity [75]. It appears that ISO-induced oxidative stress and inflammation were suppressed by treatment with melatonin and omega-3 as both exert powerful anti-oxidants and anti-inflammatory properties [67,74]. Protection of mitochondria seems to be crucial considering that these organelles (in addition to sarcoplasmic reticulum) regulate the intracellular concentration of calcium and thus play an important role in modulation of the susceptibility of the heart to arrhythmias [16].

Fundamental actions of melatonin and omega-3 may be linked with the protection of Cx43 and its functional phosphorylated status as shown in these likewise previous studies [21,67,76]. Moreover, the current study strongly points out the impact of omega-3 and melatonin on the attenuation of pro-arrhythmic distribution (lateralization) of Cx43 associated with hypertrophy and its disordered localization associated with cardiomyocyte lesion and fibrosis. Similarly, alfa-linolenic acid (precursor of omega-3) protected the heart against ISO-induced fibrosis and hypertrophy [70]. It also counteracted the increased expressions of TGF-β and the enhanced activity of MMP-2. Chlorogenic acid, a polyphenol compound, ameliorated ISO-induced myocardial injury in rats by stabilizing mitochondrial enzymes [66].

This current study suggests that long-term administration of either melatonin or omega-3 prevented ISO-induced increase in VF susceptibility in normotensive as well as in hypertensive rats. The strain-related difference points out that anti-arrhythmic efficacy might be more pronounced in subjects suffering from a deficiency of these endogenous compounds [67,77,78]. Despite missing evidence about possible anti-oxidant and/or anti-inflammatory actions of melatonin and omega-3 in the initial period of ISO application, we believe that both compounds were efficient in suppressing redox stress. We have shown that even 60 days after ISO withdrawal there was still a mild increase of SOD1 and SOD2 levels in melatonin- and omega-3-treated Wistar and SHR hearts.

## 5. Conclusions

In conclusion, the benefit of both melatonin and omega-3 most likely underlie their anti-oxidant and anti-inflammatory actions in the initial stage of ISO administration. It may result in a reduction of ISO-induced adverse signaling pathways, including pro-arrhythmic myocardial alterations, i.e., fibrosis via targeting TGF-β1/SMAD pathways and lateralization of Cx43 via normalization of Ser368-P-Cx43/PKCε pathway. These effects may fundamentally contribute to the anti-arrhythmic properties of melatonin and omega-3 in the catecholamine-stressed heart as indicated by an increased threshold to induce VF in both normotensive and hypertensive rats.

## Figures and Tables

**Table 1:** Primary antibodies used for Western blot analysis.

Antibody	Dilution	Host	Type	Supplier/# Catalogue
anti-Cx43	1:5000	Rabbit	Polyclonal	Sigma-Aldrich, St.Louis, MO, USA, #C6219
anti-phospho-serine 368-Cx43	1:1000	Rabbit	Polyclonal	Santa Cruz Biotechnology, Dallas, TX, USA, #sc-101660
anti-PKC-epsilon	1:2000	Rabbit	Polyclonal	Santa Cruz Biotechnology, Dallas, TX, USA, #sc-214
anti-TGF-β	1:1000	Rabbit	Polyclonal	Sigma-Aldrich, St.Louis, MO, USA, #SAB4502954
anti-SMAD2/3	1:1000	Rabbit	Polyclonal	Cell Signaling Technology, Danvers, MA, USA, #3102
anti-Collagen I	1:1000	Rabbit	Polyclonal	Abcam Inc.,Toronto, ON, Canada, #ab34710
anti-Keap1	1:500	Goat	Polyclonal	Abcam Inc.,Toronto, ON, Canada, #ab118285
anti-SOD-1	1:1000	Rabbit	Polyclonal	Santa Cruz Biotechnology, Dallas, TX, USA, #sc-11407
anti-SOD-2	1:1000	Rabbit	Polyclonal	Santa Cruz Biotechnology, Dallas, TX, USA, #sc-30080
anti-GAPDH	1:1000	Rabbit	polyclonal	Santa Cruz Biotechnology, Dallas, TX, USA#sc-25778

**Table 2:** General characteristics of experimental rats.

Variables	W	Wi	Wim	Wio	S	Si	Sim	Sio
**BP (mmHg)**	114 ± 17	118 ± 11	120 ± 25	140 ± 19	203 ± 24 *	190 ± 16	219 ± 41	185 ± 25
**BW (g)**	360 ± 18	359 ± 17	331 ± 36 *	369 ± 31	307 ± 29 *	322 ± 20	313 ± 17	313 ± 20
**HW (g)**	0.95 ± 0.26	1.26 ± 0.23 *	0.96 ± 0.15 ^•^	1.02± 0.15 ^•^	1.33± 0.15 *	1.40 ± 0.20	1.34 ± 0.21	1.24 ± 0.14
**LVW (g)**	0.72 ± 0.09	0,95 ± 0.22 *	0.72 ± 0.07 ^•^	0.73 ± 0.13 ^•^	1.00 ± 0.14 *	1.08 ± 0.14	0.96 ± 0.18	0.84 ± 0.26 ^•^
**EPI (g)**	5.15 ± 0.40	5.17 ± 0.82	4.64 ± 1.32	5.27 ± 0.77	3.38 ± 0.34 *	3.19 ± 0.63	2.99 ± 0.53	2.98 ± 0.26
**RET (g)**	3.18 ± 0.61	3.43 ± 0.67	3.02 ± 0.98	3.99 ± 1.10	4.01 ± 0.76	3.39 ± 1.04	2.91 ± 0.49 ^#^	3.15 ± 0.42
**EPI/BW (%)**	100 ± 7.5	101 ± 16	97 ± 25	100 ± 12	78 ± 10 *	70 ± 14	67 ± 13	67 ± 6.0
**RET/BW (%)**	100 ± 20	108 ± 22	102 ± 27	123 ± 34	149 ± 32 *	118 ± 31 ^#^	106 ± 18 ^#^	114 ± 16 ^#^

**Note:** W-Wistar rats; Wi-isoproterenol injured W; Wim-melatonin treated Wi; Wio-omacor treated Wi; S-spontaneously hypertensive rats; Si-isoproterenol injured S; Sim-melatonin treated Si; Sio-omacor treated Si; BP-Blood pressure; BW-body weight; HW-heart weight; LVW-left ventricular weight; EPI-epididymal adipose tissue; RET-retroperitoneal adipose tissue. Values are the mean ± SD of 16 rats in each group. * *p* < 0.05 compared with W; ^#^ *p* < 0.05 compared with S; ^•^ *p* < 0.05 compared to Wi vs. Wim, Wio/Si vs. Sim, Sio.

**Table 3:** Baseline values of the isolated perfused heart of experimental rats.

Variables	W	Wi	Wim	Wio	Sc	Si	Sim	Sio
**HR (beats/min)**	261 ± 33	254 ± 37	259 ± 5	222 ± 33	252 ± 18	256 ± 50	247 ± 22	234 ± 35
**CF (mL/min)**	9.90 ± 0.79	15.77 ± 3.21 *	14.20 ± 4.80	15.47 ± 4.43 *	12.63 ± 0.89	19.19 ± 1.08 ^#^	16.65 ± 0.99	13.9 ± 0.14 ^•^
**LVP (mmHg)**	14.07 ± 3.27	15.77 ± 3.21	16.77 ± 3.55	16.14 ± 2.49	13.98 ± 2.25	21.07 ± 3.46 ^#^	17.84 ± 3.08	18.23 ± 1.92
**+(dP/dt)max**	327 ± 15	470 ± 127 *	458 ± 88	497 ± 8 *	291 ± 13	604 ± 75 ^#^	564 ± 85 ^#^	630 ± 81 ^#^
**−(dP/dt)max**	222 ± 6	246 ± 74	253 ± 2	260 ± 35	199 ± 59	328 ± 39 ^#^	314 ± 40 ^#^	364 ± 35 ^#^

Note: W-Wistar rats; Wi-isoproterenol injured W; Wim-melatonin treated Wi; Wio-omacor treated Wi; S-spontaneously hypertensive rats; Si-isoproterenol injured S; Sim-melatonin treated Si; Sio-omacor treated Si; HR-heart rate; CF-coronary flow; LVP-left ventricular pressure; +dP/dtmax-index of contractility; −dP/dtmax-index of relaxation. Values are the mean ± SD of 6 rats in each group. * *p* < 0.05 compared with W; ^#^ *p* < 0.05 compared with S; ^•^ *p* < 0.05 compared to Wi vs. Wim, Wio/Si vs. Sim, Sio.
